# Thermoelectric and phonon transport properties of two-dimensional IV–VI compounds

**DOI:** 10.1038/s41598-017-00598-7

**Published:** 2017-03-30

**Authors:** Aamir Shafique, Young-Han Shin

**Affiliations:** Department of Physics, University of Ulsan, Ulsan, 44610 Republic of Korea

## Abstract

We explore the thermoelectric and phonon transport properties of two-dimensional monochalcogenides (SnSe, SnS, GeSe, and GeS) using density functional theory combined with Boltzmann transport theory. We studied the electronic structures, Seebeck coefficients, electrical conductivities, lattice thermal conductivities, and figures of merit of these two-dimensional materials, which showed that the thermoelectric performance of monolayer of these compounds is improved in comparison compared to their bulk phases. High figures of merit (*ZT*) are predicted for SnSe (*ZT* = 2.63, 2.46), SnS (*ZT* = 1.75, 1.88), GeSe (*ZT* = 1.99, 1.73), and GeS (*ZT* = 1.85, 1.29) at 700 K along armchair and zigzag directions, respectively. Phonon dispersion calculations confirm the dynamical stability of these compounds. The calculated lattice thermal conductivities are low while the electrical conductivities and Seebeck coefficients are high. Thus, the properties of the monolayers show high potential toward thermoelectric applications.

## Introduction

Renewable energy is a very important field due to the insufficiency of natural energy source and global warming^1^. One of the best renewable energy sources is waste heat, which can be converted into electricity via the Seebeck effect^2, 3^. The performance of thermoelectric materials is measured by a dimensionless quantity *ZT* called the figure of merit^4, 5^:

1 $$ZT=\frac{\sigma {S}^{2}}{\kappa }T$$

where *σ*, *S*, *κ*, and *T* are electrical conductivity, Seebeck coefficient, thermal conductivity, and temperature, respectively. The lattice thermal conductivity *κ*_*l*_ and the electronic thermal conductivity *κ*_*e*_ are included in the thermal conductivity *κ* in eq. 1. A large Seebeck coefficient, large electrical conductivity, and low thermal conductivity are needed for high thermoelectric performance, but a low amount of charge carrier is required to improve the Seebeck coefficient, which reduces the electrical conductivity^6^.

Improving *ZT* has been a big challenge, and different approaches have been used. Semiconductors composed of heavy elements such as Zn_4_Sb_3_, PbTe and BiSb have been used to reduce the thermal conductivity^7–9^. Point defects (R_1−*y*_ Fe_4−*x*_ Co_*x*_ Sb_12_ and Ce_*y*_ Fe_*x*_ Co_4−*x*_ Sb_12_) have been produced to decrease the lattice thermal conductivity and the optimized electrical conductivity^10, 11^. Some bulk complex materials also show very good thermoelectric performance such as filled skutterudites (La_0.9_ Fe_3_ CoSb_12_), half-Heusler alloys (ZrCoSn_*x*_ Sb_1−*x*_), and clathrates (Sr_8_ Ga_16_ Ge_30_) because of their low thermal conductivity and high periodicity in the crystal structure^12–16^. Zhao *et al*. recently reported that bulk SnSe is a very good thermoelectric material with a *ZT* of 2.6 at 973 K^17^. It was theoretically predicted that bulk SnS, GeSe, and GeS would also show very good thermoelectric performance^18^.

One of the efficient methods to increase *ZT* is reducing the dimensionality of the material, which increases the Seebeck coefficient due to the increased density of states near the Fermi level^19–21^. It is reported that the reduction in dimensionality enhances the energy storage and conversion^22, 23^, the *ZT* of bulk Bi_2_ Te_3_ is improved 13 times by converting into the quantum well. Fei *et al*. and Cheng *et al*. reported that a bismuth monolayer and phosphorene showed very promising thermoelectric properties^24, 25^.

We studied two-dimensional SnSe, SnS, GeSe, and GeS materials for thermoelectric applications. Monolayers of these materials have already been experimentally synthesized, and they have band gaps less than 2 eV^26–29^. They have been recently reported to have low lattice thermal conductivity as well^30^, which is a requirement for efficient thermoelectric materials. Group IV–VI compounds in bulk form have very good thermoelectric efficiency and a simple orthorhombic SnSe crystal was reported to have outstanding thermoelectricity^17, 31, 32^. It was recently discovered that even a monolayer of SnSe shows optimal thermoelectric properties^33^, which motivateed us to study the thermoelectric properties of monolayer IV–VI compounds SnSe, SnS, GeSe, and GeS.

## Results and Discussions

**Figure 1:**
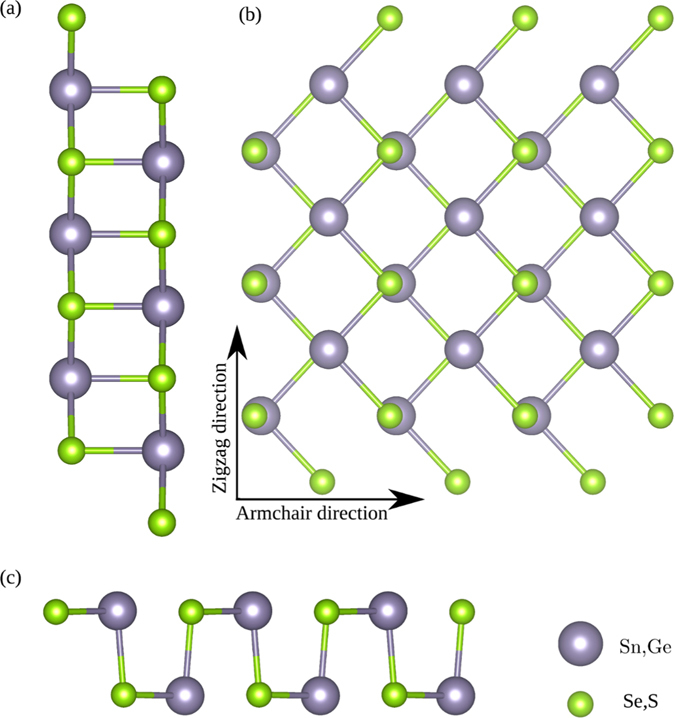
Crystal structure of two-dimensional monochalcogenides SnSe, SnS, GeSe, and GeS. (**a**) Side view perpendicular to the zigzag direction, (**b**) top view, (**c**) side view perpendicular to the armchair direction.

Bulk SnSe, SnS, GeSe, and GeS have an orthorhombic crystal structure with the space group *Pnma*(62), while their monolayer analogs have the space group *Pmn*2_1_(31) (see Fig. 1). The structures are optimized with a large vacuum space of 15 Åin the *z*-direction until the forces on each atom become zero. The optimized lattice parameters are given in Table 1, and they are in good agreement with previous reports^34, 35^.

**Table 1:** Calculated lattice parmeters and band gaps of SnSe, SnS, GeSe, and GeS. The values in the parentheses are from the refs 18 and 34.

Composition	Lattice parameter (Å)		Band gap (eV)	
	Monolayer		Monolayer	Bulk
	*a*	*b*	GGA	GGA
SnSe	4.46 (4.41)	4.29 (4.27)	0.99 (1.00)	0.65 (0.69)
SnS	4.31 (4.26)	4.07 (4.06)	1.42 (1.52)	0.86 (0.91)
GeSe	4.41 (4.38)	3.99 (3.95)	1.16 (1.22)	0.83 (0.87)
GeS	4.48 (4.43)	3.70 (3.67)	1.71 (1.70)	1.18 (1.25)

**Figure 2:**
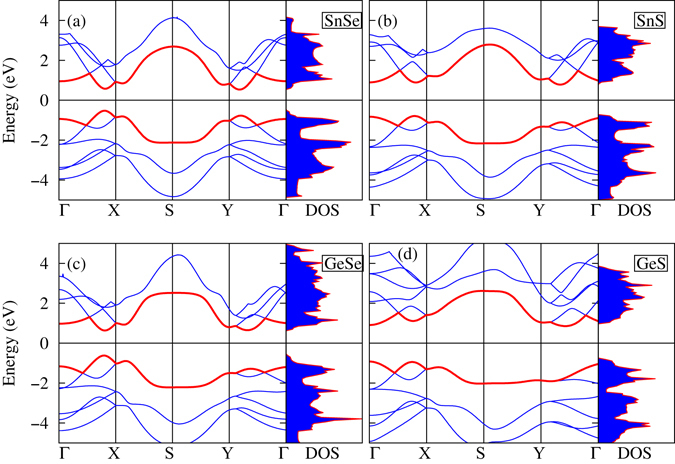
Band structures along the high-symmetry *k*-points Γ, *X*, *S*, and *Y* and density of states of (**a**) SnSe, (**b**) SnS, (**c**) GeSe, and (**d**) GeS.

Electronic structures are very important for understanding the thermoelectric behavior of materials. The band gaps of SnSe, SnS, GeSe, and GeS are calculated using the exchange-correlation functional within a generalized gradient approximation (GGA), as shown in Table 1. The GGA functional calculations show indirect band gaps for SnSe, SnS, and GeS and a direct band gap for GeSe, as shown in Fig. 2. Density of states (DOS) of the SnS and GeS monolayers has sharp peaks near conduction band minima and valence band maxima as shown in Fig. 2(b,d), which may enhance the Seebeck coefficient. All these monolayers have band gaps less than 2 eV, which suggests that they can be used as thermoelectric materials. It is very difficult to get the optimal value of the *ZT* for wide band gap materials because heavy doping is required.

The carrier mobility (*μ*) of the group IV–VI monolayers is calculated in order to get relaxation time (*τ*). Our method to calculate the mobility is based on deformation potential theory used extensively to calculate carrier mobility andrelaxation time of two-dimensional materials^36–39^, the expression to calculate the mobility is given by^36, 39, 40^:

2 $$\mu =\frac{e{\hslash }^{3}{C}^{2D}}{{k}_{B}T{m}^{\ast }{m}_{d}{E}_{1}^{2}}$$

where *C* ^2*D*^ is the two-dimensional elastic constant determined by fitting the energy-strain curve to quadratic polynomial (see Fig. S1), our calculated values for *C* ^2*D*^ are consistent with previous reported values^41^. *T* represents the temperature, *m* ^*^ is the effective mass in the transport direction, and *m*_*d*_ is calculated as *m*_*d*_ = $\sqrt{{m}_{x}{m}_{y}}$. Here *m*_*x*_ and *m*_*y*_ are the effective masses along armchair and zigzag directions. *E*_1_ is the deformation potential constant defined by *E*_1_ = $\partial {E}_{{edge}}/\partial \delta$, where *E*_*edge*_ is the conduction band minima (CBM) and *δ* is uniaxial strain. The shift in CBM by applying uniaxial strain is shown in Supplementary Fig. S2. The relaxation time is evaluated from mobility using the following relation:

3 $$\tau =\frac{{m}^{\ast }\mu }{e}$$

The calculated *E*_1_, *C*_2*D*_, *m* ^*^ and temperature-dependent *μ* and *τ* are tabulated in Table 2. We found an anisotropic behaviour in mobility and relaxation time for these monolayer. The carrier mobility of SnSe is in a good agreement with ref. 38. The GeS has the highest carrier mobility and relaxation time along the armchair direction among these four monolayer due to the low deformation constant and the low effective mass.

**Table 2:** Deformation potential constant (*E*_1_), two dimensional elastic constant (*C* ^2*D*^), effective mass (*m* ^*^), carrier mobility (*μ*) and relaxation time (*τ*) at 300 K, 500 K, and 700 K, in the armchair and zigzag directions of the group IV–VI compounds.

Composition	Direction	*E*_1_ (eV)	*C* ^2*D*^ (N/m)	*m* ^*^ (*m*_*e*_)	*T* = 300 K		*T* = 500 K		*T* = 700 K	
					*μ* (cm^2^ V^−1^ s^−1^)	*τ* (fs)	*μ* (cm^2^ V^−1^ s^−1^)	*τ* (fs)	*μ* (cm^2^ V^−1^ s^−1^)	*τ* (fs)
SnSe	Armchair	3.32	12.46	0.15	1035.84	88.35	621.50	53.01	443.93	37.86
	Zigzag	4.70	24.82	0.16	965.23	87.82	579.13	52.69	413.60	37.63
SnS	Armchair	2.78	14.08	0.24	623.54	85.10	374.13	51.06	267.23	36.47
	Zigzag	4.27	26.05	0.28	419.10	66.73	251.48	40.04	179.63	28.60
GeSe	Armchair	2.49	12.11	0.27	541.24	83.10	324.74	49.86	231.96	35.61
	Zigzag	3.91	28.35	0.30	465.01	79.33	279.01	47.59	199.29	33.99
GeS	Armchair	2.37	13.89	0.19	1045.40	112.95	627.24	67.77	448.03	48.40
	Zigzag	3.70	33.40	0.37	529.57	111.43	317.73	66.86	226.95	47.75

**Figure 3:**
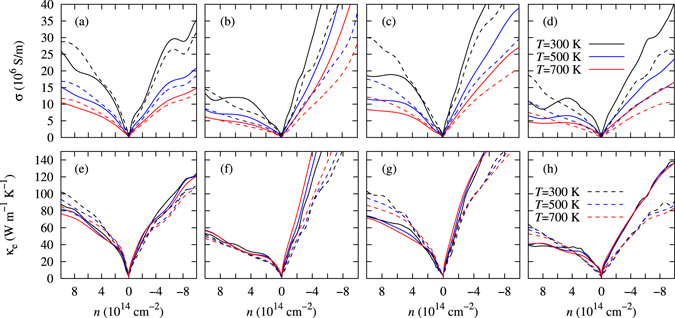
Electrical (*σ*) and electronic thermal (*κ*_*e*_) conductivities for the (**a**,**e**) SnSe, (**b**,**f**) SnS, (**c**,**g**) GeSe, and (**d**,**h**) GeS along the armchair (solid lines) and zigzag (dashed lines) directions at 300 K, 500 K, and 700 K.

The thermoelectric properties of SnSe, SnS, GeSe, and GeS are calculated using the Boltzmann transport equation for electrons under a constant scattering time. Boltztrap code calculates-relaxation-time dependent electrical conductivity (*σ*/*τ*) and electronic thermal conductivity (*κ*_*e*_/*τ*). Since there is no experimental data available for the electrical conductivity to calculate the exact value of the relaxation time of these monolayers, deformation potential theory is used to predict the temperature-dependent relaxation time for each material as compiled in Table 2. The electrical (*σ*) and electronic thermal (*κ*_*e*_) conductivities are plotted as a function of carrier concentration (*n*) in Fig. 3(a–d) and (e–g) respectively, at 300 K, 500 K, and 700 K along armchair and zigzag directions. The carrier concentration shows the doping (positive values for *p*-type doping and negative values for *n*-type doping). The electrical and thermal conductivities increase by increasing carrier concentration. When the Fermi level occurs in the middle band gap region, the conductivities are increased slowly with respect to the carrier concentration and when it moves down into the valence band (for *p*-type) or moves up into conduction band (for *n*-type), the conductivities are increased quickly. GeSe has the highest electrical conductivity of 69.85 × 10^6^ S/m at *n* = −8.9 × 10^14^/cm^2^ in the *n*-type doping among these compounds. The band gap is higher for the monolayer than bulk (see Table 1). The monolayers have lower electrical conductivity than the bulk due to the increase in the band gaps.

**Figure 4:**
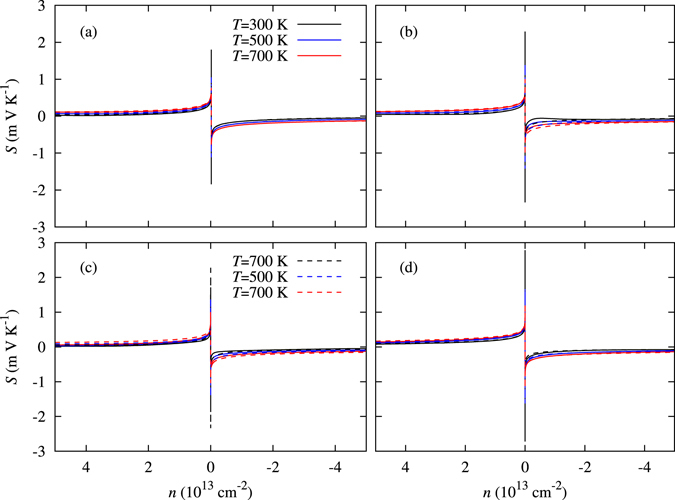
Calculated Seebeck coefficients (*S*) as a function of the carrier concentration (*n*) along the armchair (solid lines) and zigzag (dashed lines) directions at 300 K, 500 K, and 700 K for the group IV–VI monolayers (**a**) SnSe, (**b**) SnS, (**c**) GeSe, and (**d**) GeS.

The Seebeck coefficients are calculated as a function of carrier concentration at different temperatures along the armchair and zigzag direction, as shown in Fig. 4. As the temperature decreases, the Seebeck coefficient also increases because of bipolar conduction^42^. The Seebeck coefficients of the two-dimensional monochalcogenides are two times greater than those of the bulk material as shown in the Table 3. This results from, the increase in the band gaps and the density of states near the Fermi level. The GeS has the largest Seebeck coefficient of 2810 *μ* VK^−1^ at 300 K because of the large band gap and the flatness in the band structure. The Seebeck coefficient (*S*) is calculated with the expression,

4 $$S=\frac{{\int }_{-\infty }^{\infty }dE\,g(E)(E-\mu )(-\frac{\partial f(E,\mu ,T)}{\partial E})}{T{\int }_{-\infty }^{\infty }dE\,g(E)(\frac{\partial f(E,\mu ,T)}{\partial E})},$$

where *E*, *g*(*E*), *f*(*E*, *μ*, *T*), *μ*, and *T* are energy, transport function, Fermi function, chemical potential, and temperature, respectively^43^. The transport function is:

5 $$g(E)=N(E){v}^{2}(E)\tau (E),$$

where *N*(*E*) is the density of states, *v*(*E*) is the Fermi velocity and *τ*(*E*) is the scattering time^43^. The Seebeck coefficient changes dramatically near the Fermi level because of the term $\frac{\partial f}{\partial E}$ in Eq. 4, which behaves like a Dirac delta function.

**Table 3:** The largest values of Seebeck coefficients (*S*) of bulk and monolayer SnSe, SnS, GeSe, and GeS at 300 K (unit: *μ* VK^−1^).

Composition	SnSe	SnS	GeSe	GeS
Bulk^1^	990	1260	1240	2000
Monolayer	1750	2380	1960	2810

^1^Ref. 18.

**Figure 5:**
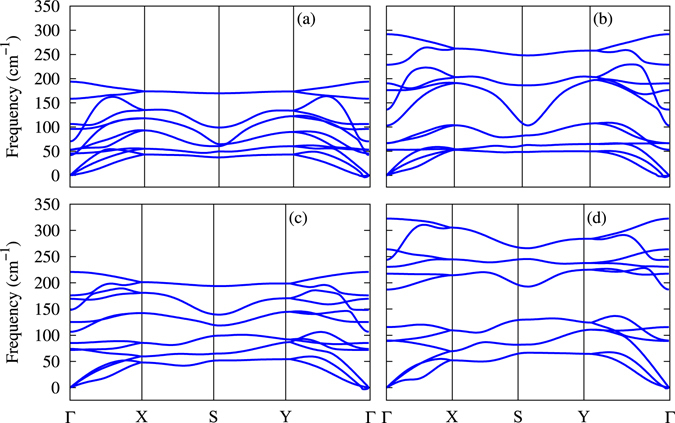
Phonon dispersions along high symmetry *k*-points for (**a**) SnSe, (**b**) SnS, (**c**) GeSe, and (**d**) GeS.

Phonon dispersions of SnSe, GeSe, SnS, and GeS were computed to examine the thermal stability using density functional perturbation theory^44^, as shown in Fig. 5. There is no imaginary line in the dispersion curves, which means that these materials are vibrationally stable. There are twelve modes of vibrations: three loweer modes that are acoustic (a transverse acoustic mode, a longitudinal acoustic mode, and a flexural acoustic mode), and the others are optical modes. The flexural acoustic mode is an out-of-plane transverse acoustic mode similar to other two-dimensional materials like graphene, phosphorene, and stanene, quadratic near Γ point^45–47^. The flexural mode vibrational direction is exactly perpendicular to the plane. It is an important mode in order to understand thermal and mechanical properties of two-dimensional materials.

**Figure 6:**
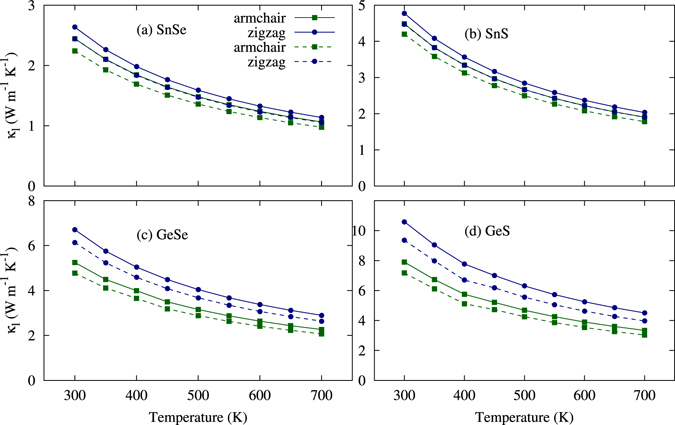
Lattice thermal conductivity (*κ*_*l*_) for the group IV–VI monolayers are calculated as a function of the temperature using iterative (solid lines) and SMRTA (dashed lines) method.

The lattice conductivities are calculated by solving the Boltzmann transport equation for phonons (BTEP) using the iterative method and the relaxation time approximation (RTA). The iterative method exactly solves the BTEP, while RTA is a good approximation for low conductivity compounds. The results in Fig. 6 show good agreement with recently reported results^30^. All four of the materials have very low lattice thermal conductivity compared to other two-dimensional materials like graphene, phosphorene, and monolayers of MoSe_2_ and WSe_2_, and are comparable to bismuth monolayer as shown in Table 4 ^25, 45, 46, 49^. We also found the different lattice thermal conductivity along the armchair and zigzag directions. Because of the heavy masses of Sn and Se, SnSe has the lowest lattice conductivity of 2.44 Wm^−1^ K^−1^ and 2.63 Wm^−1^ K^−1^ at room temperature along the armchair and zigzag directions, respectively.

**Table 4:** Comparison of lattice thermal conductivities *κ*_*l*_ of group IV–VI monolayers with other two-dimensional materials at room temperature.

Material	*κ*_1_ (Wm^−1^ K^−1^)		
	Monolayer		Bulk
	zigzag	armchair	
Graphene	2200^45^	2200^45^	2000^48^
Phoshorene	30.1^46^	13.6^46^	—
Bi monolayer	3.8^25^	3.8^25^	—
MoSe_2_	70^49^	70^49^	40^49^
WSe_2_	42^49^	42^49^	35^49^
SnSe	2.6	2.4	0.32^18^
SnS	4.7	4.4	0.45^18^
GeSe	6.7	5.2	0.39^18^
GeS	10.5	7.8	0.52^18^

According to glass dynamical theory, the lattice thermal conductivity is calculated as ${\kappa }_{l}=1/3{C}_{v}l{v}_{s}$, where *C*_*v*_ is the heat capacity, *l* is the mean free path, and *v*_*s*_ is the sound velocity. As the temperature increases, the lattice softens and the stiffness decreases, which reduces the sound velocity and hence the lattice thermal conductivity^50^. This trend is shown in Fig. 6.

**Figure 7:**
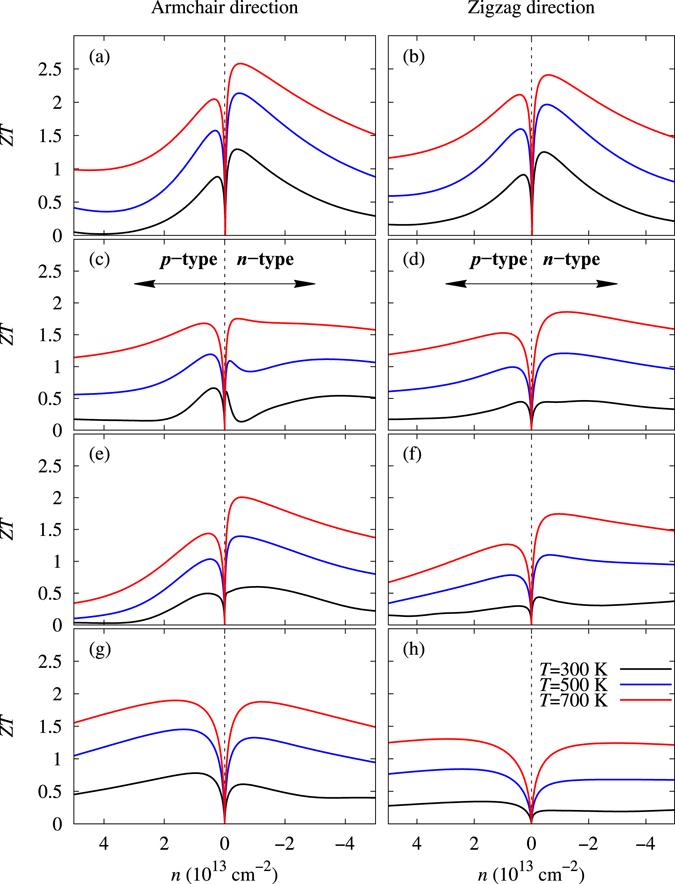
Calculated Figures of merit (*ZT*) as a function of the carrier concentration (*n*) for the monolayer of (**a**,**b**) SnSe, (**c**,**d**) SnS, (**e**,**f**) GeSe, and (**g**,**h**) GeS along armchair and zigzag directions at temperature 300 K, 500 K, and 700 K.

Finally, using the Seebeck coefficient and the electrical and thermal conductivities, we calculated *ZT* as a function of the carrier concentration along the armchair and zigzag directions at 300 K, 500 K, and 700 K, as shown in Fig. 7. These monolayers had very high *ZT*. SnSe had the highest *ZT* of 2.63 along the armchair direction at 700 K because of the high electrical conductivity and Seebeck coefficient and the low lattice thermal conductivity. In the case SnS, a high *ZT* of 1.88 is predicted along the zigzag direction.

### Conclusion

We analyzed the structural, electronic, thermoelectric, and phonon-transport properties of the two-dimensional monochalcogenide compounds SnSe, SnS, GeSe, and GeS using density functional theory combined with Boltzmann transport theory for electrons and phonons. These compounds are energetically and vibrationally stable, and SnSe, SnS, and GeS have indirect band gaps while GeSe has a direct band gap. The Seebeck coefficients of these two-dimensional materials are two times larger than those of their bulk structures, and two-dimensional GeS has the largest Seebeck coefficient of 2810 *μ* V K^−1^ at room temperature. These monolayer materials have very low lattice thermal conductivities in comparison to other two-dimensional materials. *ZT* of SnSe, GeSe and GeS along the armchair direction was 2.63, 1.99, and 1.85, respectively, while that of *ZT* of SnS along the zigzag direction was 1.88. These *ZT* values are higher than those of their bulk analogs. Hence, the materials are very promising for thermoelectric applications.

## Methods

Our calculations are based on density functional theory combined with Boltzmann transport theory and were performed using the Vienna Ab initio Simulation Package (VASP) and the Boltztrap code^51–53^. The generalized gradient approximation proposed by Perdew-Burke-Ernzerhof was chosen as an electronic exchange correlation functional^54^. The vdW-DF scheme is used to include the van der Waals interaction^55^. A Monkhorst mesh of 10 × 10 × 1 *k*-points is used for lattice optimization and 450 eV is used as a plane wave cutoff energy. Structures are optimized until the Hellmann–Feynman force on each atom is less than 0.001 eV/Å. A vacuum region of 15 Å in the *z*-direction is produced to avoid the interaction between periodic images.

Thermoelectric properties were computed by solving the Boltzmann transport equation under a constant relaxation time (*τ*) and a rigid band approximation performed in the Boltztrap code^53^. We used a very dense *k*-point mesh of 60 × 60 × 1 to obtain a convergent density of states. The Seebeck coefficient *S*(*T*, *n*), electrical (*σ*/*τ*) and electronic thermal (*κ*_*e*_/*τ*) conductivities devided by the relaxation time were calculated. Boltztrap code used Wiedemann-Franz law to calculate electronic thermal conductivty from electrical conductivity.

To calculate the lattice thermal conductivity (*κ*_*l*_), we used the ShengBTE code^56^. The second-order (harmonic) and the third-order (anharmonic) interatomic force constants (IFCs) are required to calculate lattice thermal conductivity. In order to calculate the second-order IFCs, we used the Phonopy code with a supercell of 5 × 5 × 1 and a *k*-mesh of 10 × 10 × 1. For the third-order IFCs, a supercell of 4 × 4 × 1 was used with the of interactions up to the 15th nearest neighbors^57–59^.

## Electronic supplementary material

Supplementary information
