# Supplementary material for: Thermoelectric and phonon transport properties of two-dimensional IV–VI compounds
Source: Sci Rep. 2017 Mar 30;7:506. doi: 10.1038/s41598-017-00598-7 (PMC5428725; doi:10.1038/s41598-017-00598-7)
Supplement: Supplementary file 1 — Supplementary information [file 41598_2017_598_MOESM1_ESM.pdf]

Thermoelectric and phonon transport properties of two-dimensional IV–VI compounds (supplementry information)

Aamir Shafique and Young-Han Shin*
Department of Physics, University of Ulsan, Ulsan 44610, Republic of Korea

* To whom correspondence should be addressed: hoponpop@ulsan.ac.kr

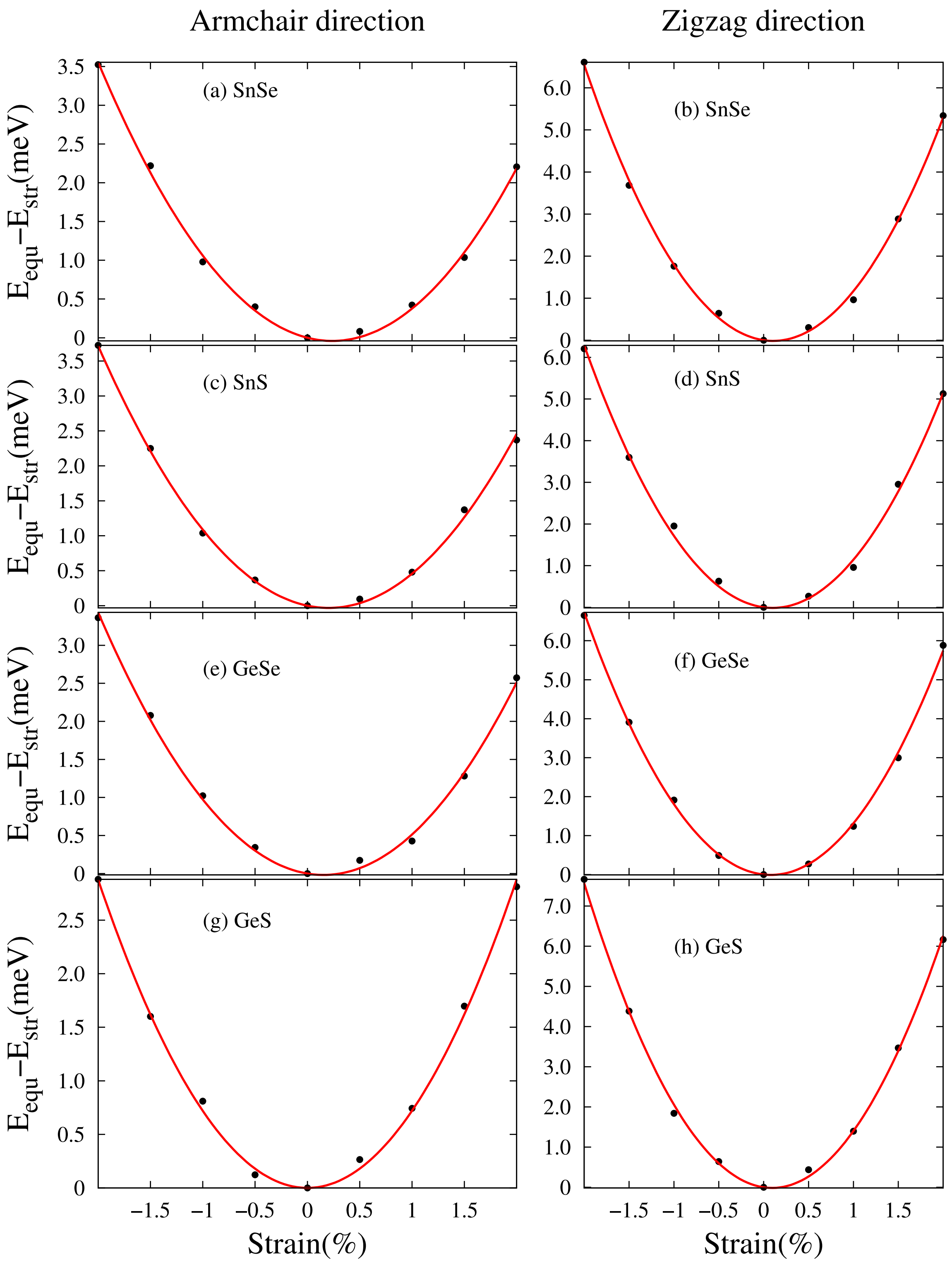

Figure 1. Elastic constants are calculated by fitting the energy difference ($E_{\rm equ} - E_{\rm str}$ where $E_{\rm equ}$ is the total energy in the equilibrium state and $E_{\rm str}$ is the total energy in the strained state) to quadratic polynomial functions for (a, b) SnSe, (c, d) SnS, (e, f) GeSe, and (g, h) GeS monolayers along armchair and zigzag directions.

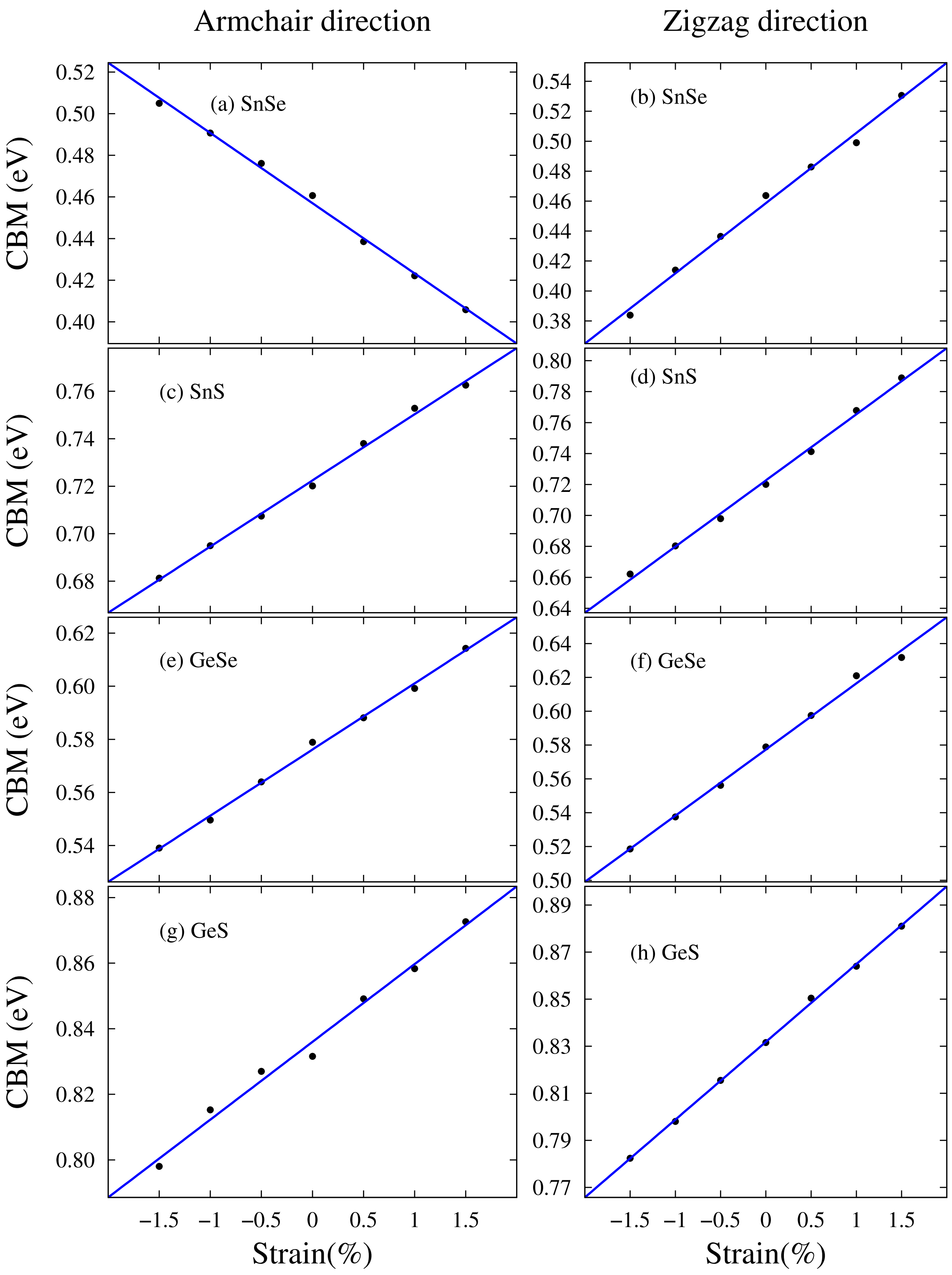

Figure 2. Calculated deformation constants for group IV-VI compounds: (a, b) SnSe, (c, d) SnS, (e, f) GeSe, and (g, h) GeS monolayers. The conduction band minima (CBM) at uniaxial strains are fitted to straight lines.
